# Lung Cancer with Brain Metastasis—Treatment Strategies and Molecular Characteristics

**DOI:** 10.3390/jcm13237371

**Published:** 2024-12-03

**Authors:** Shuai Wang, Matan Uriel, Haiying Cheng

**Affiliations:** Department of Oncology, Montefiore Medical Center/Albert Einstein College of Medicine, Bronx, New York, NY 10461, USA

**Keywords:** lung cancer, brain metastases, immunotherapy, targeted therapy

## Abstract

Lung cancer is a leading cause of brain metastases (BMs), with 10–20% of patients with non-small cell lung cancer (NSCLC) presenting with BMs at diagnosis and 25–50% developing them over the course of their disease. Historically, BMs have posed significant therapeutic challenges, partly due to the blood brain barrier (BBB), which restricts drug penetration to the central nervous system. Consequently, BMs were initially managed with local treatments, including surgical resection, stereotactic radiosurgery, and whole brain radiation therapy. In recent years, however, systemic treatments for BMs have advanced significantly, particularly with the development of molecularly-targeted therapies and immunotherapies. The discovery of driver mutations and the development of novel tyrosine kinase inhibitors (TKIs) have yielded encouraging intracranial responses in NSCLC patients with actionable genetic alterations (e.g., *EGFR*, *ALK*, *ROS1*). Genomic profiling has also suggested genetic heterogeneity between BMs and primary sites. Immunotherapies, alone or in combination with other treatments, have demonstrated promising results in NSCLC with BMs, although most clinical trials have included only selected patients with asymptomatic or previously treated BMs. In this review, we discuss the molecular and immune characteristics of NSCLC with BMs, analyze intracranial efficacy findings from clinical trials, and explore treatment strategies for lung cancer patients with BMs.

## 1. Introduction

Brain metastases (BMs) have been a significant cause of morbidity and mortality in cancer patients, affecting up to 45% of all cancer patients with systemic disease and accounting for approximately 20% of cancer related deaths [1,2]. The prognosis for patients diagnosed with BMs has remained notoriously poor, with a 5-year overall survival (OS) of 2.4% across all cancer types [3]. Additionally, central nervous system (CNS) metastases and their treatments have been shown to significantly reduce quality of life (QoL) for patients with BMs, who often have limited survival [4]. Lung cancer continues to be the leading cause of cancer related death in the United States [5], and is also one of the primary contributors to the development of BMs, accounting for 40–50% of cases [1,6]. Approximately 20% of patients with non-small cell lung cancer (NSCLC) will present with BMs at the time of diagnosis and 25–50% will develop BMs during their disease courses [6].

**Figure 1:**
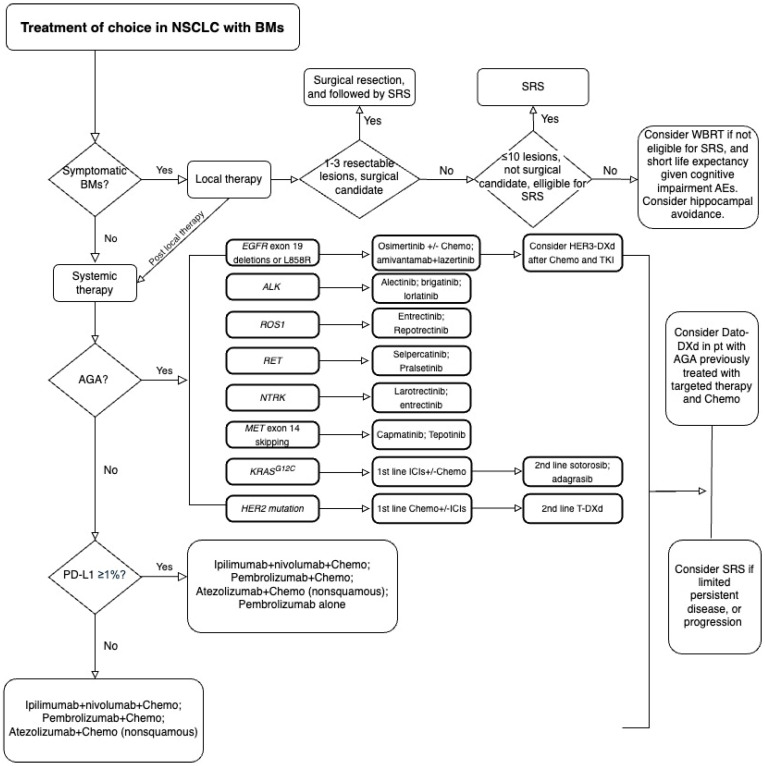
Treatment of choice in non-small cell lung cancer with brain metastases. NSCLC: non-small cell lung cancer; BMs: brain metastases; SRS: stereotactic radiosurgery; WBRT: whole brain radiation therapy; AEs: adverse events; AGA: actionable genetic alteration; Chemo: platinum-based chemotherapy; PD-L1: programmed death-ligand 1; ICIs: immune checkpoint inhibitors; *EGFR*: epidermal growth factor receptor; *ALK*: anaplastic lymphoma kinase; *ROS1*: ROS proto-oncogene 1; *RET*: Ret protooncogene; *NTRK*: neurotrophic tyrosine receptor kinase; *MET*: mesenchymal–epithelial transition; *KRAS^G12C^*: kirsten rat sarcoma viral oncogene homolog G12C; *HER2*: human epidermal growth factor receptor 2; T-DXd: trastuzumab deruxtecan; HER3-DXd: patritumab deruxtecan; Dato-DXd: datopotamab deruxtecan.

Historically, the brain has been a difficult therapeutic target in metastatic NSCLC due to the blood brain barrier (BBB) and the limited penetration of drugs into the CNS. Hence, BM therapies were initially approached with local treatments which included surgical resection, stereotactic radiosurgery (SRS), and whole brain radiation therapy (WBRT). However, recent years have shown significant improvement of systemic treatment modalities such as targeted therapies and immunotherapies [6]. Immunotherapies alone or in combination with other therapies have shown promising results in NSCLC with BMs. With advancements in the discovery of driver mutations and new tyrosine kinase inhibitor (TKI) developments, targeted therapies also showed encouraging results of intracranial activity (IC) in NSCLC with actionable genetic alterations (AGAs) in clinical trials. To date, local therapies continue to be the cornerstone of treatment for brain metastases (BMs), particularly in neurologically symptomatic patients. However, the roles of upfront TKIs and immunotherapies in BMs are evolving, as reflected in the guidelines [7,8]. The current treatment of choice in NSCLC with BMs is described in Figure 1. Genomic profiling in some studies suggested heterogeneity of genetic features between BMs and primary tumor or other metastatic sites. In this review, we focus on treatment strategies of BMs in lung cancer by comparing different IC responses in clinical trials and discussing the molecular characteristics of NSCLC with BMs.

## 2. Local Therapy

Local therapies, including neurosurgical resection and SRS, should be considered for neurologically symptomatic patients or with limited number of BMs (approximately one to three BMs). Solitary BMs management was first investigated in the 1990s and studies showed that the combination of surgery and post-operative WBRT decreased local recurrence and prolonged survival compared to either monotherapy alone [9,10]. The current standard of care (SOC) for resectable and limited number of BMs is surgical resection followed by post-operative SRS. This was adopted following a few clinical trials. In a randomized controlled phase III trial, 132 patients with one to three BMs were recruited. Surgical resection followed by SRS was found to have higher 12-month freedom of local recurrence compared to surgical resection alone (72% vs. 43%; HR 0.46, 95% CI 0.24–0.88), but there was no difference in OS [11]. Another randomized phase III trial involving 194 patients with BMs compared post-operative SRS to post-operative WBRT. SRS was found to result in longer cognitive free deterioration survival (3.7 m vs. 3.0 m; HR 0.47, 95% CI 0.35–0.63), but shorter time to CNS disease progression (median 6.4 m vs. 27.5 m; HR 2.45, 95% CI 1.62–3.72). However, OS was not significantly different between the two groups [12]. For patients who are not surgical candidates, SRS is the treatment of choice. Two phase III clinical trials have compared SRS alone or with WBRT. Both trials showed that SRS alone did not show any change in OS [13,14]. SRS alone showed less cognitive decline (45.5% vs. 94.1%) and better QoL at three months compared to WBRT [13]. Additionally, shorter time to CNS disease progression and higher need for salvage RT were observed in SRS alone [14]. Due to these clinical trials and others, WBRT has fallen out of favor due to its potential long term neurocognitive effects in the management of BMs in NSCLC.

## 3. Systemic Therapy

### 3.1. Chemotherapy

Historically, chemotherapy has shown limited benefits and low response rates in the treatment of BMs. It is likely due to the low penetration through the BBB. However macroscopic metastatic disease often causes disruption to the BBB and higher bioavailability of the drug to that area [6]. The response rates for first-line chemotherapy in advanced NSCLC with BMs ranged from 20–50% in previous trials [15]. In the immunotherapy era, chemotherapy in combination with immunotherapy is the first-line treatment for advanced NSCLC without AGAs.

### 3.2. Immunotherapy

Immune check point inhibitors (ICIs) with or without chemotherapy have been the SOC of first-line treatment in advanced NSCLC without AGAs and those with *KRAS* mutations. It is an emerging option for the treatment of BMs in NSCLC as survival benefits have been observed in the subgroup with BMs in several landmark clinical trials [16,17]. Treatment modalities including immunotherapy alone, dual immunotherapy, and immunotherapy in combination with chemotherapy will be discussed below.

#### 3.2.1. Immunotherapy Alone

In a pooled analysis of KEYNOTE 001, 010, 024, and 042, the outcomes of patients with advanced NSCLC with positive PD-L1 and BMs were investigated [18]. The pooled data compared pembrolizumab monotherapy to chemotherapy in both previously treated or treatment-naïve settings. A total of 3170 patients were included in this study, and 293 (9.2%) had baseline BMs. Among the patients with BMs, pembrolizumab compared to chemotherapy revealed favorable OS (HR 0.67; 95% CI 0.44–1.02 in PD-L1 ≥ 50%; HR 0.83; 95% CI 0.62–1.10 in PD-L ≥ 1%) and progression free survival (PFS) (HR 0.70; 95% CI 0.47–1.03 in PD-L1 ≥ 50%; HR 0.96; 95% CI 0.73–1.25 in PD-L ≥ 1%), though the differences were not statistically significant [18]. Similar results were also seen in other phase III clinical trials with different immunotherapies. In the subgroup analysis study from the phase III OAK trial, atezolizumab was compared to docetaxel in advanced NSCLC, with or without BMs, in the second-line setting [19]. There was 14% of patients that had baseline BMs, and among this cohort, median OS was numerically longer with atezolizumab than docetaxel (16.0 m vs. 11.9 m; HR 0.74; 95% CI 0.49–1.13) [19]. Further evidence of direct IC response was provided from a phase II clinical trial that enrolled 42 advanced NSCLC patients with untreated BMs or progression following local therapy with pembrolizumab. The study demonstrated that among the patients with PD-L1-positive (TPS ≥ 1%) scores, 29.7% (95% CI 15.9–47.0) achieved IC responses, whereas no IC response was observed in the PD-L1 negative cohort [20].

#### 3.2.2. Immunotherapy Included Combination Therapy

Immunotherapy combined with platinum-based chemotherapy has been investigated in different ICIs. A pooled subgroup analysis study evaluated the efficacy of first-line pembrolizumab plus chemotherapy versus chemotherapy alone for advanced NSCLC, with or without BMs, from KEYNOTE 021 (nonsquamous), 189 (nonsquamous), and 407 (squamous) [21]. Among the 1298 patients that were enrolled, 171 (13.2%) had BMs at baseline. In patients with BMs, median OS was significantly longer with pembrolizumab plus chemotherapy versus chemotherapy alone (18.8 m, 95% CI 13.8–25.9 vs. 7.6 m, 95% CI 5.4–10.9; HR 0.48; 95% CI 0.32–0.70), and significantly longer PFS was also observed in the combination cohort versus chemotherapy alone (6.9 m, 95% CI 5.7–8.9 vs. 4.1 m, 95% CI 2.3–4.6; HR 0.44; 95% CI 0.31–0.62). Importantly, the PFS benefits of combination therapy in the BMs cohort were significant across all PD-L1 expression groups (<1%, 1–49%, ≥50%) [21]. A recent single arm phase II trial (n = 40) evaluated atezolizumab plus chemotherapy in advanced nonsquamous NSCLC with asymptomatic treated or untreated BMs. The median systemic PFS was 8.9 months (95% CI 6.7–13.8), while the IC PFS was 6.9 months (95% CI 4.7–11.9). Of note, in this study, IC ORR and systemic ORR were similar at around 45% and no differences in ORR were observed across different PD-L1 groups (<1%, 1–49%, ≥50%) [22].

Inspired by IC responses of dual immunotherapies in metastatic melanoma [23], the CheckMate 227 trial was the first phase III trial to explore IC activity in advanced NSCLC. Though ipilimumab in combination with nivolumab compared to chemotherapy did not yield significant IC PFS benefit (IC mPFS 8.6 m vs. 8.7 m; HR 0.8; 95% CI 0.50–1.27), the 4-year IC PFS showed a numerically higher rate in the dual immunotherapies arm versus chemotherapy arm (28% vs. 7%) [24]. Dual immunotherapies plus chemotherapy was subsequently evaluated and showed IC efficacy in NSCLC with BMs. In the randomized phase III CheckMate 9LA trial, first-line ipilimumab and nivolumab in combination with chemotherapy compared to chemotherapy alone showed improved survival. A subgroup analysis showed that among the 14% of patients who had baseline BMs, dual immunotherapies and chemotherapy combination yielded superior median OS over chemotherapy alone (19.3 m, 95% CI 12.3–23.9 vs. 6.8 m, 95% CI 4.7–9.7; HR 0.45; 95% CI 0.29–0.70). Similarly, PFS benefit was observed in the combination arm over chemotherapy arm. IC activity was also assessed with an improved IC ORR in the combination arm (39.2% vs. 20.0%). Also, IC median PFS was more favorable in the combination arm than the chemotherapy arm (11.4 m, 95% CI 8.4–18.6 vs. 4.6 m, 95% CI 3.2–5.7; HR 0.42; 95% CI 0.26–0.68). In addition, fewer patients developed new BMs, favoring dual immunotherapies plus chemotherapy (20% vs. 30%, respectively) [25].

It is important to note that most of these trials only enrolled patients with previously treated and asymptomatic BMs. Some trials enrolled asymptomatic patients with untreated BMs and showed comparable OS to reported data among patients with asymptomatic and treated BMs [20,22,26]. This suggested that immunotherapy included treatment could be beneficial to this vulnerable population.

### 3.3. Targeted Therapy—TKI

Targeted therapies have become SOC for patients with advanced NSCLC with AGAs in the first-line and subsequent-line settings. The incidence of BMs varies from 20–50% across molecular subtypes, and IC responses are different based on genomic alterations and types of targeted therapies [27]. A systemic review and meta-analysis studied the genomic alterations and incidences of BMs in advanced NSCLC, and showed that the pooled prevalence of BMs at diagnosis was 28.6%, highest in *ALK* rearranged patients (34.9%), followed by those with *RET* translocations (32.2%), *KRAS* (30.2%), *ROS1* (30.1%), and *EGFR* (29.4%) [28]. In recent years, growing evidence has demonstrated the IC activity of newer-generation TKIs in advanced NSCLC with BMs. Table 1 summarizes the IC efficacy results from the major trials.

#### 3.3.1. *EGFR* TKIs

Osimertinib, a third-generation *EGFR* TKI, is one of most studied TKIs and has robust IC activity compared to early-generation TKIs (erlotinib and gefitinib). It was approved for the first-line treatment of metastatic NSCLC whose tumors have *EGFR* exon 19 deletions or exon 21 L858R mutations following the results of the FLAURA trial in 2018 [29,30]. Recently, two other combination treatments were approved by the Food and Drug Administration (FDA) for the same setting [30,31]. In the FLAURA trial, IC response rate for patients with ≥one measurable CNS lesion was 91% (95% CI 71–99) for the osimertinib cohort compared to 68% (95% CI 43–87) for those who received first-generation TKIs (gefitinib or erlotinib) [31]. The FLAURA 2 trial evaluated osimertinib with or without chemotherapy in *EGFR*-mutated (exon 19 deletions or exon 21 L858R) advanced NSCLC and revealed longer systemic PFS in the combination arm versus the osimertinib alone arm among patients with baseline BMs (median PFS 24.9 m vs. 13.8 m; HR 0.47; 95% CI 0.33–0.66) [32]. The MARIPOSA study evaluated amivantamab (*EGFR-MET* bispecific antibody) plus lazertinib (third-generation *EGFR* TKI) in previously untreated *EGFR*-mutated (exon 19 deletions or exon 21 L858R) advanced NSCLC, and showed favorable PFS in patients with BMs compared to osimertinib alone (HR 0.69; 95% 0.53–0.92) [33]. For *EGFR* T790M mutation, which is a major cause of acquired *EGFR* TKI resistance, both osimertinib (IC ORR 66.7%; 95% CI 54.3–79.1) and lazertinib (IC ORR 85.7%; 95% CI 59.8–100) have shown strong IC responses in early phase clinical trials [51,52]. Osimertinib also demonstrated activity against leptomeningeal disease in a phase I trial with a leptomeningeal objective response of 62% and 43% by a neuroradiological blinded center independent review and an investigator, respectively [53].

#### 3.3.2. *ALK* TKIs

Historically, BMs have been a challenge for *ALK* rearranged NSCLC. Though compared to first-generation *ALK* TKI crizotinib, second- and third-generation *ALK* TKIs (alectinib, brigatinib, ceritinib, and lorlatinib) have shown improved IC responses. The ALEX trial compared alectinib to crizotinib in previously untreated *ALK*-positive NSCLC. In patients with baseline BMs, alectinib showed improved median PFS compared to crizotinib (25.4 m vs. 7.4 m; HR 0.37; 95% CI 0.23–0.58) [34]. In the ALTA-1L trial, brigatinib was compared to crizotinib in *ALK*-positive advanced NSCLC who were *ALK* TKI-naïve and asymptomatic or stable BMs patients were enrolled. The trial showed beneficial IC ORR in the brigatinib cohort (78% vs. 26%; HR 11.7; 95% CI 2.15–63.27; *p* = 0.0014) and 3-year IC PFS (31% vs. 9%; HR 0.29; 95% CI 0.17–0.51) [35,36]. The phase III CROWN study demonstrated that lorlatinib compared to crizotinib had better PFS and IC activity for treatment-naïve patients with *ALK*-positive advanced NSCLC. Among patients with baseline BMs, the mPFS in the lorlatinib arm was not reached (NR) compared to 6.0 months in the crizotinib arm (HR 0.08; 95% CI 0.04–0.19). The IC ORR was higher with lorlatinib than with crizotinib (60% vs. 11%, respectively), with a complete response (CR) rate of 49% in the lorlatinib group versus 5% in the crizotinib group [37]. Of note, in the 5-year follow up study, the median time to IC progression was NR, and the probability of IC progression free was 92% (95% CI 85–96) with lorlatinib at 5 years [37].

#### 3.3.3. *ROS1* TKIs

About 1–2% of NSCLC patients were found to have *ROS1* rearrangement and up to 40% of patients with *ROS1*-positive metastatic disease presented with BMs [6]. There are three *ROS1* TKIs that are approved by the FDA for first-line treatment in advanced NSCLC with *ROS1* fusion with crizotinib being the first TKI approved for this indication. However, de novo or acquired resistance has been found in crizotinib which has limited its CNS activity [54]. Entrectinib showed better IC response. In an integrated analysis of three phase I or II clinical trials (ALKA-372-001, STARTRK-1, STARTRK-2), entrectinib has shown IC ORR of 52.2% (95% CI 37.0–67.1) among patients with baseline BMs. The median IC PFS was 8.3 months (95% CI 6.4–15.7). However, for patients who previous received crizotinib, entrectinib provided only a modest response, suggesting limited activity against G2032R–mutant *ROS1* fusions [38]. Next-generation repotrectinib was investigated in the phase I/II TRIDENT-1 trial. The study demonstrated a response rate of 79% (95% CI 68–88%) in patients who had not previously received a *ROS1* TKI, and a response rate of 38% (95% CI 25–52%) in patients who had received one *ROS1* TKI but had never undergone chemotherapy. In the subgroup of patients with baseline BMs, IC responses were observed in 89% of those who were *ROS1* TKI-naïve and in 38% of those who had received one *ROS1* TKI [39].

#### 3.3.4. *KRAS*^G12C^ TKIs

Despite *KRAS*^G12C^ mutations occur in approximately 10–13% of patients with advanced NSCLC, effective treatment options have been limited historically. Currently, sotorasib and adagrasib are the two FDA approved drugs for advanced NSCLC patients harboring the *KRAS*^G12C^ mutation in the second- and subsequent-line settings. In the CodeBreaK 200 trial, the first phase III trial evaluating a *KRAS*^G12C^ inhibitor in NSCLC, sotorasib demonstrated a longer median time to CNS recurrence compared to docetaxel in advanced NSCLC patients with *KRAS*^G12C^ and baseline BMs who had previously received chemotherapy and immunotherapy (15.8 m vs. 10.5 m; HR 0.52; 95% 0.26–1.0) [40], although the difference was not significantly different [40]. Adagrasib was investigated firstly in KRISTAL-1, a phase I/II trial in previously treated advanced NSCLC patients with *KRAS*^G12C^ mutation [41]. In the phase II study part, among the 42 patients with baseline BMs, 33% (95% CI 18.0–51.8) had an IC response and the median IC PFS was 5.4 months (95% CI 3.3–11.6) [41]. Another subgroup study of the phase Ib KRISTAL-1 trial evaluated IC activity of adagrasib in 25 patients with previously treated advanced NSCLC with *KRAS*^G12C^ and untreated BMs. Adagrasib revealed that the IC ORR was 42% (95% CI 20.3–66.5), and median IC PFS was 5.4 months (95% CI 2.7-not evaluable) [55]. Most recently, the efficacy and safety outcomes in patients with and without BMs from the phase III KRISTAL-12 trial were reported in *The European Society for Medical Oncology (ESMO)* 2024, and demonstrated that among 114 (25.2%) patients who had baseline BMs, adagrasib compared to docetaxel provided longer mPFS (4.4 m vs 2.9 m; HR 0.7; 95% CI 0.4–1.2) and greater systemic ORR (21% vs. 1%) [42,43].

#### 3.3.5. Other TKIs

In *RET* fusion-positive patients, both selpercatinib [44,45] and pralsetinib [46] showed IC activities from phase I/II trials with an IC ORR of 82% (95% CI 60–95)^50^ and 70% (95% CI 35–93) [46], respectively. In *NTRK* fusion-positive patients, larotrectinib [47], entrectinib [48], and repotrectinib [56] are currently FDA approved for advanced NSCLC. Larotrectinib and entrectinib showed positive IC responses [47,48]. Though the final report for IC activity of repotrectinib in the TRIDENT-1 study among *NTRK* fusion-positive patients is still pending, it is promising based on robust IC activity of repotrectinib among *ROS1* fusion patients [39]. For *MET* exon 14 skipping (*MET*ex14) patients, both capmatinib [49] and tepotinib [50] showed IC activity with IC ORR of 57% and 66.7%, respectively. The data of BMs in *BRAF*^V600E^-mutated NSCLC are sparse, though the combination of *BRAF* and *MEK* inhibitors suggested IC activity in open label phase II trials. In the PHAROS trial, 98 patients with advanced NSCLC with *BRAF*^V600E^ were treated with encorafenib plus binimetinib, and eight patients had baseline BMs. All four patients who were treatment-naïve had either CR or partial response (PR), while none of the four previously treated patients had a response [57]. Dabrafenib plus trametinib yielded a systemic ORR of more than 60% in an open label phase II study in advanced NSCLC patients with *BRAF*^V600E^. However, only one patient with asymptomatic BMs (1/57) was enrolled and had a non-CR/non-PD response in their brain lesion [58].

### 3.4. Antibody Drug Conjugate (ADC)

ADCs are becoming an increasingly promising approach in NSCLC. So far, trastuzumab deruxtecan (T-DXd) is the only FDA approved ADC targeting *ERBB2* (*HER2*) mutations in previously treated advanced NSCLC. A post hoc analysis study from DESTINY-LUNG01 and DESTINY-LUNG02 trials evaluated IC activity of T-DXd and showed an IC ORR of 25.0% (T-DXd dose 5.4 mg/kg) and 18.5% (T-DXd does 6.4 mg/kg) [59,60]. The FDA ultimately approved the 5.4 mg/kg dose due to the increased incidence of interstitial lung disease/pneumonitis observed at higher doses. T-DXd was also investigated in previously treated NSCLC patients with HER2 expression (immunohistochemistry +2 or +3) in the phase 2 DESTINY-Lung01 trial. The result showed a systemic ORR of 26.5% and 34.1% in cohort 1 (6.4 mg/kg) and cohort 1A (5.4 mg/kg), respectively [61]. Although patients with BMs were enrolled, no specific subgroup data for BMs or IC activity were reported. However, considering the IC ORR of 73.7% observed in HER2-positive breast cancer [62], a similar effect in NSCLC could be inferred. Other ADCs that also showed promising IC activities including patritumab deruxtecan (HER3-DXd) targeting HER-3 for third-line treatment in *EGFR*-mutated advanced NSCLC [63], and datopotamab deruxtecan (Dato-DXd) targeting Trop-2 for previously treated advanced NSCLC with AGAs, etc. [64]. Several main trials investigating ADCs in NSCLC with BMs are listed in Table 2.

## 4. Genetic and Molecular Characteristic of NSCLC with BMs

The molecular characteristics of BMs in NSCLC have been investigated in prior studies and are often different from those of the primary tumor site, reflecting the tumor revolution and the distinct microenvironment of the brain [65,66,67,68,69,70]. An earlier study with whole-exome sequencing of 86 matched BMs, primary tumors, and normal tissues observed the branched evolution, where the metastatic sites and primary tumor all shared a common ancestor and continued to evolve independently [65]. The study detected alterations associated with sensitivity to *PI3K/AKT/mTOR, CDK*, and *HER2/EGFR* inhibitors in the BMs [65]. A comprehensive genomic profiling of 3035 NSCLC BM cases versus unmatched primary tumors showed higher rates of several targetable genetic alternations in BMs compared to primary sites, including *ALK* fusion, *KRAS*^G12C^ mutations and *MET* amplifications [66]. Another study compared the genomic features of 233 NSCLC patients with resected BMs and matched samples (47 primary tumor, 42 extracranial metastasis), and found enriched *CDKN2A/B* deletions and cell cycle pathways in BMs samples [67]. The study also demonstrated notable clinico-genomic correlations including *EGFR* mutations in leptomeningeal disease and *MYC* amplifications in multifocal regional brain progression. Other examples including higher prevalence of *TP53* mutations, *EGFR* mutations, and *TERT* amplifications in lung adenocarcinoma with BMs [68], and higher frequency of *MYC*, *YAP1*, and *MMP13* amplification in BMs of NSCLC [70]. Table 3 summaries trials evaluating molecular characteristics of in BMs of advanced NSCLC.

## 5. Discussion

Management of BMs in NSCLC has long been difficult due to the protective nature of the BBB preventing medications from penetrating the CNS. Cancer cells can disrupt the BBB by surface ligands and promote extravasation of cancer cells into the brain parenchyma, leading to the formation of a remodeled brain–tumor barrier (BTB). This altered barrier, marked by dysfunctional astrocytes, pericytes, and endothelial connections, is more permeable than an intact BBB. This increased permeability allows entry of not only cancer cells, but also immune cells and small molecules (e.g., TKIs) into the CNS, thereby enabling some therapeutic approaches in NSCLC with BMs including ICIs and targeted therapy etc. [71].

In advanced NSCLC with BMs and without AGAs, the SOC currently includes frontline ICI, either alone or in combination with platinum-based chemotherapy or dual ICIs. Although IC efficacy has been demonstrated in various trials, it is observed in fewer than 50% of patients [16,17,18,19,20,21,22,24,25,26], highlighting an unmet need in this patient group. Along with the BBB, the lack of lymphatic drainage is another factor that contributes to the brain being an immune-privileged organ. The tumor microenvironment (TME), including factors such as the upregulation and infiltration of cytotoxic T lymphocytes from the periphery and the density of tumor-infiltrating lymphocytes (TILs), plays a crucial role in the treatment of NSCLC with BMs [72,73]. Current and future strategies to overcome ICI refractoriness in NSCLC with BMs requires multifaceted approaches that increase the ICI penetration of the BBB and target the TME.

The combination of ICI with radiotherapy addresses both approaches and has shown a superior survival outcome with numerically higher IC responses in a meta-analysis [74]. However the sequence of ICI and radiotherapy in asymptomatic BMs remains unclear. ICI with targeted therapy is also investigated to enhance the ICI efficacy. This includes bispecific antibody ivonescimab targeting PD-1 and vascular endothelial growth factor (VEGF). At the recent World Conference on Lung Cancer (WCLC), the results of the phase III HARMONI 2 trial were reported, showing that first-line ivonescimab compared to pembrolizumab had led to significantly longer median PFS (11.14 m vs. 5.82 m; HR 0.51; 95% CI 0.38–0.69; *p* < 0.0001) in advanced NSCLC patients with a positive PD-L1 score. The PFS benefit was consistent in NSCLC patients both with and without BMs [75]. Though subgroup analysis on IC activity from this trial is not yet available, two previous phase II trials have showed promising IC activity for ivonescimab, with a combined IC ORR of 34%, and a median IC PFS of 19.3 months [76]. The aforementioned ivonescimab studies were conducted solely in China, and further validation in other countries is necessary for broader application. Other strategies and future directions involve targeting immune checkpoint proteins beyond PD-(L)1 and CTLA-4. These include targeting molecules such as lymphocyte activation gene-3 (LAG-3), T cell immunoglobulin and mucin-domain-containing protein-3 (TIM-3), and T cell immunoreceptor with immunoglobulin and ITIM domain (TIGIT), among others [77].

In recent years, targeted therapies, including TKIs, have shown considerable success in treating NSCLC with BMs and AGAs. Furthermore, the growing number of trials assessing ADCs and bispecific antibodies indicates that more promising treatment options are emerging for NSCLC patients with AGAs. This includes telisotuzumab-vedotin (targets c-Met) [78], and sacituzumab govitecan (targets Trop-2) [79]. Both have demonstrated promising systemic activity, though their intracranial activity still needs to be evaluated. With the increased use of genetic profiling in general and in different sample types for NSCLC, it has become evident that the genetic landscape in BMs differs from that of primary tumors and other extracranial metastatic sites [65,66,67,68,69,70]. This can result in differential responses to targeted therapy in oncogene-driven NSCLC with BMs, highlighting the need for enhanced genetic profiling of brain samples to confirm actionable targets. Challenges include the need for invasive procedures for tissue sampling, as well as the fact that some genetic alterations present in BMs are still not targetable.

Lastly, more clinical trials, especially phase III trials, are warranted to enroll NSCLC patients with untreated BMs, as this group is often excluded in existing trials, resulting in a knowledge gap about the efficacy and safety of new therapies in those with untreated or newly diagnosed BMs.

## 6. Conclusions

The therapeutic strategies for patients with advanced NSCLC and BMs are rapidly evolving, offering new opportunities for patients with historically limited options. Advances in immunotherapies, targeted therapies, ADCs, and radiotherapy techniques, as well as combination therapies are providing more effective and personalized treatment approaches, improving both IC and overall outcomes. Distinct genetic profiles in BMs compared to other tissue sites indicate unique biological mechanisms driving IC progression. Developing and implementing more effective CNS-penetrating targeted therapies is crucial for managing patients with BMs from oncogene-driven lung cancers. Deciphering the genetic signatures that drive BMs is essential for identifying novel disease-specific targets and developing more effective treatments.

## Figures and Tables

**Table 1:** Clinical trials evaluating intracranial activity of TKIs in advanced NSCLC.

Study	Trial Type	Genetic Alterations	Patient N./Population/ BMs N.	BMs Eligibility Criteria	Intervention	Systemic Outcomes	IC Outcomes
FLAURA [29,30,31]	Phase III, randomized	*EGFR* exon 19 deletions or L858R	556/advanced NSCLC, first-line/128 had BMs, 41 had measurable lesions	Asymptomatic or stable BMs	1:1 ratio to osimertinib 80 mg daily or gefitinib 250 mg or erlotinib 150 mg daily	mPFS: 18.9 m vs. 10.2 m (HR 0.46; 95% CI 0.37–0.57; *p* < 0.001) mOS: 38.6 m vs. 31.8 m (HR 0.80; 95% CI 0.64–1.00; *p* = 0.046) ORR: 80% vs. 76% (OR 1.27; 95% CI 0.85–1.90; *p* = 0.24)	IC ORR: 91% vs. 68% (OR 4.6; 95% CI 0.9–34.9; *p* = 0.066) IC mPFS: NR vs. 13.9 m (HR 0.48; 95% CI 0.26–0.86; *p* = 0.014)
FLAURA 2 [32]	Phase III, randomized	*EGFR* exon 19 deletions or L858R	557/advanced NSCLC, first-line/226 had BMs	Stable BMs	1:1 ratio to osimertinib 80 mg daily + platinum-based chemotherapy or osimertinib monotherapy	mPFS: 25.5 m vs. 16.7 m (HR 0.62; 95% CI 0.49–0.79; *p* < 0.001) ORR: 83% vs. 76%	mPFS in BMs: 24.9 m vs.13.8 m (HR 0.47; 95% CI 0.33–0.66)
MARIPOSA [33]	Phase III, randomized	*EGFR* exon 19 deletions or L858R	858/advanced NSCLC, first-line/350 had BMs	Asymptomatic or stable BMs	1:1 ratio to amivantamab-lazertinib or osimertinib	mPFS: 23.7 m vs. 16.6 m (HR 0.70; 95% CI 0.58–0.85; *p* < 0.001) ORR: 86% vs. 85%	mPFS in BMs: 18.3 m vs. 13.0 m (HR 0.69; 95% CI 0.53–0.92)
ALEX [34]	Phase III randomized	*ALK*-positive	303/advanced NSCLC, first-line/122 had BMs	Asymptomatic brain or leptomeningeal metastases	1:1 ratio to alectinib or crizotinib	mPFS: 34.8 m vs. 10.9 m (HR 0.43; 95% CI 0.32–0.58)	mPFS in BMs: 25.4 m vs. 7.4 m (HR 0.37; 95% CI 0.23–0.58)
ALTA-1L [35,36]	Phase III randomized	*ALK*-positive	275/advanced NSCLC, no previous *ALK* inhibitors/96 had BMs	Asymptomatic or stable BMs	1:1 ratio to brigatinib or crizotinib	mPFS: 24.0 m vs. 11.1 m (HR 0.48; 95% CI 0.35–0.66; *p* < 0.001)	IC ORR 78% vs. 26% (OR 11.7; 95% CI 2.15–63.27; *p* = 0.0014)
CROWN [37]	Phase III, randomized	*ALK*-positive	296/advanced NSCLC, first-line/38 had BMs	Asymptomatic treated or untreated BMs	1:1 ratio to lorlatinib or crizotinib	mPFS: NR vs. 9.1 m (HR 0.19; 95% CI 0.13–0.27) ORR 81% vs. 63%	mPFS in BMs: NR vs. 6.0 m (HR 0.08; 95% CI 0.04–0.19) IC ORR: 60% vs. 11% IC mPFS: NR vs. 16.4 (0.06; 95% CI 0.03–0.12)
ALKA-372–001 STARTRK-1 STARTRK-2 [38]	Integrated analysis of phase I/II trials	*ROS1*-positive	161/locally advanced or metastatic NSCLC/56 had BMs	Asymptomatic or pretreated and controlled BMs	Entrectinib	mPFS: 15.7 m (95% CI 11.0–21.1) ORR: 67.1% (95% CI 59.3–74.2)	IC ORR: 52.2% (95% CI 37.0–67.1) IC mPFS: 8.3 m (95% CI 6.4–15.7)
TRIDENT-1 [39]	Phase I/II	*ROS1*-positive	127 in primary efficacy cohort/locally advanced or metastatic NSCLC/21 had BMs	Treated or untreated asymptomatic BMs	Repotrectinib	1st line mPFS: 34.1 (95% CI 27.4-NE) 1st line ORR: 79% (95% CI 68–88%)	1st line IC ORR: 89% (95% CI 52–100%); 2nd line IC ORR: 38% (95% CI 14–68) IC PFS at 12 m: 1st line: 91% (95% CI 83–100); 2nd line: 82% (95% CI 65–98)
CodeBreaK 200 [40]	Phase III randomized	*KRAS* ^G12C^	345/advanced NSCLC with KRAS^G12C^ who received platinum-based chemotherapy and ICI/118 had BMs	Treated, asymptomatic BMs	1:1 ratio to sotorasib or docetaxel	mPFS: 5.6 m vs. 4.5 m (HR 0.66 (95% CI 0.51–0.86; *p* = 0.0017) ORR: 28.1% vs.13.2%	median CNS recurrence: 15.8 m vs. 10.5 m (HR 0.52; 95% CI 0.26–1.0)
KRISTAL-1 [41]	Phase II part	*KRAS* ^G12C^	116/advanced NSCLC with KRAS^G12C^ who received platinum-based chemotherapy and ICI/42 had BMs	Treated and neurologically stable BMs	Adagrasib	mPFS: 6.5 m (95% CI 4.7–8.4) mOS: 12.6 m (95% CI 9.2–19.2) ORR: 42.9% (95% CI 33.5–52.6)	IC ORR: 33% (95% CI 18.0–51.8) IC mPFS: 5.4 m (95% CI 3.3–11.6)
KRISTAL-12 [42,43]	Phase III randomized	*KRAS* ^G12C^	453/advanced NSCLC with KRAS^G12C^ who received platinum-based chemotherapy and ICI/114 had BMs	Treated, neurologically stable baseline BMs	2:1 ratio to adagrasib or docetaxel	mPFS: 5.5 m vs. 3.8 m (HR 0.58; 95% CI 0.45–0.76; *p* < 0.0001) ORR: 31.9% vs. 9.2% (OR 4.68; 95% CI 2.56–8.56; *p* < 0.0001)	mPFS in BMs: 4.4 m vs. 2.9 m (HR 0.7; 95% CI 0.4–1.2) ORR in BMs: 21% vs. 1%
LIBRETTO-001 [44,45]	Phase I/II	*RET* fusion	316/advanced NSCLC (69 treatment naïve, 247 previously treated with chemotherapy)/80 had BMs	Asymptomatic or neurologically stable for ≥2 weeks	Selpercatinib	Treatment-naïve patients: mPFS: 22.0 m (95% CI 13.8-NE) ORR: 84% (95% CI 73–92) Previous treated patients: mPFS 24.9 m (95% CI 19.3-NE) ORR: 61% (95% CI 55–67)	IC ORR: 82% (95% CI 60–95) IC: mPFS 13.7 m (95% CI 10.9-NE)
ARROW [46]	Phase I/II	*RET* fusion	233 advanced NSCLC (75 treatment naïve, 158 previously treated)/87 had BMs.	Excluded patients with progressive neurological symptoms or requires increasing doses of corticosteroids to control the CNS disease	Pralsetinib	Treatment-naïve patients: mPFS: 13.0 m (95% CI 9.1-NR) ORR: 79% (95% CI 59–92) Previous treated with platinum-based chemotherapy: mPFS: 16.5 m (95% CI 10.5–24.1) ORR: 59% (95% CI 50–67)	IC ORR: 70% (95% CI 35–93) IC mDOR: 10.5 m (95% CI 5.5–12.6)
NAVIGATE [47]	Phase I/II	*NTRK* fusion	20/previously treated advanced NSCLC/10 had BMs	Asymptomatic	Larotrectinib	mPFS: 35.4 m (95% CI 5.3–35.4) mOS: 40.7 m (95% CI 17.2-NE) ORR: 73% (95% CI 45–92)	Systemic ORR in BMs: 63% (95% CI 25–91)
ALKA-372-001 STARTRK-1 STARTRK-2 [48]	Integrated analysis of phase I/II trials	*NTRK* fusion	51/advanced NSCLC without previous TKI/20 had BMs	Asymptomatic or previously treated and controlled	Entrectinib	mPFS 28.0 m (95% CI 15.7–30.4) mOS 41.5 m (95% CI 30.9-NE) ORR: 62.7% (95% CI 48.1–75.9)	IC ORR: 64.3% (95% CI 35.1–87.2) IC PFS: 32.7 m (95% CI 5.9-NE)
GEOMETRY mono-*1* [49]	Phase II	*METex 14* skipping	160/advanced NSCLC (60 treatment naïve; 100 previously treated)/28 had BMs	Neurologically stable or asymptomatic BM.	Capmatinib	Treatment-naïve ORR: 68% (95% CI 55–79.7) Previously treated ORR: 44% (95% CI 34.1–54.3)	IC ORR: 57%; in patients with no previous IC RT, IC ORR: 67%
VISION [50]	Phase II	*METex 14* skipping	161/advanced NSCLC/43 had BMs	Neurologically stable and glucocorticoid dose was being tapered down, or untreated asymptomatic BM.	Tepotinib	ORR: 54.7% (95% CI 46.6–62.5) mPFS 13.8 m (95% CI 10.4-NE)	IC ORR: 66.7% (95% CI 38.4–88.2) IC mPFS: 20.9 m (95% CI 5.7-NE)

NSCLC: non-small cell lung cancer; TKIs: tyrosine kinase inhibitors; BMs: brain metastases; IC: intracranial; ORR: objective response rate; PFS: progression free survival; OS: overall survival; NR: not reached; NE: not evaluable; RT: radiotherapy; *EGFR*: epidermal growth factor receptor; *ALK*: anaplastic lymphoma kinase; *ROS1*: ROS proto-oncogene 1; *RET*: Ret proto-oncogene; *NTRK*: neurotrophic tyrosine receptor kinase; *MET*: mesenchymal–epithelial transition; *KRAS^G12C^*: kirsten rat sarcoma viral oncogene homolog G12C.

**Table 2:** Clinical trials evaluating intracranial activity of ADCs in advanced NSCLC with BMs.

Study	Trial Type	Target	Patient N./Population/ BMs N.	BMs Eligibility Criteria	Intervention	Systemic Outcomes	IC Outcomes
DESTINY-LUNG01; DESTINY-LUNG02 [59,60]	Pooled analysis of phase II trials	*HER2* mutation	5.4 mg/kg: 102 advanced NSCLC previously treated with chemotherapy/32 had BMs 6.4 mg/kg: 141 advanced NSCLC previously treated with chemotherapy/27 had BMs	Stable, asymptomatic BMs	DESTINY-LUNG01 cohort 2: 6.4 mg/kg T-DXd DESTINY-LUNG02: 2:1 ratio to 5.4 mg/kg and 6.4 mg/kg T-DXd	DESTINY-LUNG02: ORR: 49.0% (95% CI 39.0–59.1) for 5.4 mg/kg and 56.0% (95% CI 41.3–70.0) for 6.4 mg/kg mPFS: 9.9 m (95% CI 7.4-NE) for 5.4 mg/kg and 15.4 m (95% CI 8.3-NE) for 6.4 mg/kg mOS: 19.5 m (95% CI 13.6-NE) for 5.4 mg/kg and NE (95% CI 12.1-NE) for 6.4 mg/kg	Systemic ORR in BMs: 46.9% (5.4 mg/kg) and 50.0% (6.4 mg/kg) IC ORR: 25.0% (5.4 mg/kg) and 18.5% (6.4 mg/kg) IC DOR: 4.6 m (5.4 mg/kg) and 7.2 m (6.4 mg/kg)
HERTHENA-Lung01 [63]	Phase II randomized	*HER3*	277 advanced *EGFR* (exon 19 deletions or L858R) NSCLC previously treated with *EGFR* TKI and chemotherapy/115 had BMs, and 30 BMs evaluated	Clinically inactive or treated BMs that were asymptomatic	Fixed dose arm received HER3-DXd 5.6 mg/kg; Uptitration arm received HER3-DXd 3.2→ 4.8 → 6.4 mg/kg	ORR: 29.8% (23.9–36.2) mPFS: 5.5 m (95% CI 5.1–5.9) mOS: 11.9 m (95% CI 11.2–13.1)	IC ORR: 33% (17.3–52.8) IC DOR: 8.4 m (95% CI 5.8–9.2)
TROPION-LUNG05 [64]	Phase II	*TROP2*	137 advanced NSCLC with AGAs previously treated with targeted therapy and chemotherapy/53 had BMs	Clinically stable BMs	Dato-DXd	ORR: 35.8% (27.8–44.4) mDOR 7.0 m	IC ORR: 22% (95% CI 6–48) IC DOR: 5.5 m (95% CI 3.4-NE)

ADC: antibody drug conjugate; NSCLC: non-small cell lung cancer; BMs: brain metastases; IC: intracranial; ORR: objective response rate; PFS: progression free survival; OS: overall survival; DOR: duration of response; NE: not evaluable; T-DXd: trastuzumab deruxtecan; HER3-DXd: patritumab deruxtecan; Dato-DXd: datopotamab deruxtecan; AGAs: actionable genetic alterations.

**Table 3:** Studies evaluating genetic and molecular characteristics in BMs of advanced NSCLC.

Study	Year	N. of Patients	Histology	Finding
Brastianos PK et al., Cancer Discovery [65]	2015	86 (38 lung)	Different origins, among lung mostly adenocarcinoma and squamous cell	Metastatic site and primary tumor show common ancestor but metastatic site shows branched evolution; 53% of cases had clinically relevant genetic alteration in BMs which were not detected in primary site. Different BM sites were genetically homogenous. Detected alterations with associated sensitivity to *PI3K/AKT/mTOR*, *CDK*, or *HER2* inhibitors in BMs
Paik PK et al., Cancer Discovery [69]	2015	79	Squamous Cell	*PI3K* aberrant tumors had greater incidence of BMs (27% vs. 0% in others, *p* < 0.001); BMs exhibited a high degree of genetic heterogeneity and evidence of clonal differences vs. primary sites
Shih et al., Nature genetics [70]	2020	73 with BMs and 503 in control arm	Adenocarcinoma	Increased amplification of *MYC, YAP1*, and *MMP13* and increased deletions of *CDKN2A/B* genes were associated with a higher BM rate
Huang et al., The oncologist [66]	2022	3035	NSCLC	Higher rates of targetable genetic alterations, including *ALK* fusion, *KRAS*^G12C^ mutations, and *MET* amplifications in BMs compared to primary site
Nguen et al., Cell [68]	2022	25,000	Adenocarcinoma	Higher rates of *TP53* and *EGFR* mutations and *TERT* amplification in lung adenocarcinoma patients with BMs
Skakodub et al., Nature Communications [67]	2023	233	NSCLC	Increased *CDKN2A/B* deletions and cell cycle pathway alterations in BMs. Increased *EGFR* mutations in leptomeningeal disease and *MYC* amplifications in multifocal regional brain progression

NSCLC: non-small cell lung cancer; BMs: brain metastases.

## Data Availability

No new data were created.
